# Underdiagnosis in Background of Emerging Public Health Challenges Related to Peri-Implant Diseases: An Interventional Split-Mouth Study

**DOI:** 10.3390/ijerph20010477

**Published:** 2022-12-28

**Authors:** Boris Djuran, Zoran Tatic, Neda Perunovic, Natasa Pejcic, Jovana Vukovic, Aleksandra Petkovic-Curcin, Danilo Vojvodic, Mia Rakic

**Affiliations:** 1 Department of Oral Implantology, Military Medical Academy, 11000 Belgrade, Serbia; 2 Faculty of Dental Medicine, University of Belgrade, 11000 Belgrade, Serbia; 3 Biocell Hospital, 11070 Beograd, Serbia; 4 Faculty of Biology, University of Belgrade, 11000 Belgrade, Serbia; 5 Institute for Medical Research, Military Medical Academy, 11000 Belgrade, Serbia; 6 ETEP (Etiology and Therapy of Periodontal Diseases) Research Group, University Complutense of Madrid, 28040 Madrid, Spain; 7 Institute for Biological Research “Sinisa Stankovic”, University of Belgrade, 11000 Belgrade, Serbia

**Keywords:** peri-implant diseases, peri-implantitis, peri-implant mucositis, biomarker, treatment, bone loss, precision medicine, early diagnosis, public health

## Abstract

Peri-implant diseases are an emerging public health problem, and it’s considered that limitations of standard diagnostics play the role herein. The study objective was the estimation of pathological bone resorption at clinical and biological level in patients with peri-implant mucositis (PIM) and peri-implantitis (PI) before and 6 months after standard treatment and to compare them with healthy controls (HC). The split-mouth interventional study included 60 patients affected with PIM or PI. Patients that also presented at least one more HC were enrolled in the study and underwent standard non-surgical and surgical treatment, respectively. Standard clinical parameters and soluble levels of RANKL were measured in peri-implant crevicular fluid baseline and 6 months following treatment. Clinical parameters and RANKL significantly decreased following treatment in PIM and PI. However, bleeding on probing and probing depth remained significantly increased when compared to HC. RANKL answered requests for biomarker of peri-implant diseases, its baseline levels were significantly increased in PIM and PI, they decreased following treatment and reached HC in peri-implantitis, while in PIM RANKL remained significantly increased. Presence of pathological bone resorption in patients lacked its clinical signs, and respective persistence following treatment suggest the need for biomarker-supported diagnosis for timely diagnosis of peri-implantitis and appropriate orientation of respective management strategies.

## 1. Introduction

Peri-implant diseases are qualified as a growing problem in dentistry and is an emerging public health issue associated with substantially negative socio-economic impact and negative oral and systemic consequences [1]. The first factor qualifying peri-implant diseases as emerging public health problem is its increasing prevalence currently ranging between 18.5–56.6% at the patient levels and 12.8–27.9% at the implant level [2,3]. These respective rates gain tremendous effects given the continuous growth of the implant market worldwide. The second qualifying reason is the lack of predictive treatment, which represents a far more concerning aspect of peri-implant disease [4,5]. This underlies a high diseases recurrence, and is the first cause of implant failure, while negatively impacting implant treatment cost-effectiveness. Thus, the early diagnosis of patient responsiveness to treatment remains of critical importance for the timely adjustment of a treatment plan and is related to prevention of further disease progression requiring more complex approach of already unpredictable treatments of peri-implant diseases [6,7]. The diagnostic accuracy of standard clinical parameters on implants is substantially due to the lack of periodontal ligament, while the variability in implant designs, implantation techniques and inter-individual peri-implant bone remodeling out-profile additionally compromise their respective reproducibility [8,9]. Subsequently, clinical diagnosis on implants relies on composite and comparable assessment of multiple parameters, while the lack of their respective initial records expectedly decreases the accuracy of implant diagnosis. This particularly concerns early diagnosis of pathological bone resorption within peri-implantitis onset. Subsequently, clinical distinction of peri-implant mucositis (PIM), the condition where peri-implant inflammation remains contained within soft peri-implant tissues, from early peri-implantitis additionally implies the spread of inflammatory process to the bone, which is particularly challenging [10]. In contrast to periodontal disease, where gingivitis can be easily discriminated form periodontitis, the conversion of PIM into peri-implantitis still remains unclear, which represents a major clinical concern given the asymptomatic course of peri-implantitis associated with accelerating non-linear pattern of progression [11].

Bone loss is a critical diagnostic parameter in all stages of clinical decision-making on dental implants, starting from discrimination between reversible and irreversible state of destruction, over treatment planning and prognosis, until evaluation of the treatment success and monitoring of long-term treatment outcomes. Peri-implant bone loss is routinely evaluated by means of a composite assessment of clinical and radiological parameters [10]. However, the variability in peri-implant bone remodeling and implant designs affect standardization of clinical and radiological parameters on implants [8,9]. This is why the comparative approach represents the most reliable approach in the assessment of peri-implant bone remodeling. Subsequently, the lack of initial bone level values bears a challenge for accurate diagnosis of pathological bone resorption [12]. Given the complex peri-implant biology, the actual underlying specificities of peri-implant diseases compared to periodontal diseases and subsequently decreased capacity of clinical strategies adopted from periodontology on implants, the personalization of management strategies for peri-implant diseases remains to be a subject of strongest recommendation [13,14]. It is expected that biomarkers may contribute to an early diagnosis of peri-implantitis, to appropriate treatment planning with evolutive modulation for an optimal patient compliance, as well as for customization of recalls for successful maintenance of the stable treatment outcomes [15].

Bone turnover markers (BTMs) are highly responsive regulators of bone remodeling that are able to quantitatively reflect the volume of bone resorption in the real-time—even before radiological signs of osteolysis [16]. Receptor activator nuclear factor kappa-B ligand (RANKL) is an intrinsic regulator of inflammatory osteoclastogenesis and is certainly within the most repurposed BTMs in in vitro diagnostics (IVDs) that was widely investigated for clinical application in periodontology and implantology [17,18]. The interaction of RANKL and its cognate receptor RANK represents the principal regulatory pathway of osteoclast differentiation, maturation and activation [19]. In brief, RANK is located on the surfaces of pre-osteoclasts and osteoclasts, and its ligation with RANKL leads to fusion of osteoclast precursors into mature osteoclasts and upregulation of mature osteoclasts [20]. Finally, local inflammation proportionally upregulates RANKL expression and the respective rate of RANK–RANKL interaction, which is followed by concomitant amplification of osteoclastogenesis and enhanced bone resorption [21]. RANKL was confirmed as a highly specific marker of peri-implantitis, and proposed as a promising candidate marker which is capable of accurately discriminate peri-implant conditions and able to improve accuracy of respective clinical diagnosis [13,18,22].

The working hypothesis was that RANKL can accurately indicate pathological peri-implant bone resorption bellow clinical detectability within early diagnosis of peri-implantitis and estimation of the patients’ compliance to administrated treatment.

The objectives of the present study were to estimate pathological bone resorption at clinical and biological levels in patients with peri-implant mucositis (PIM) and peri-implantitis (PI) before and 6 months after standard treatment and to compare them with healthy controls (HC).

## 2. Materials and Methods

**Figure 1:**
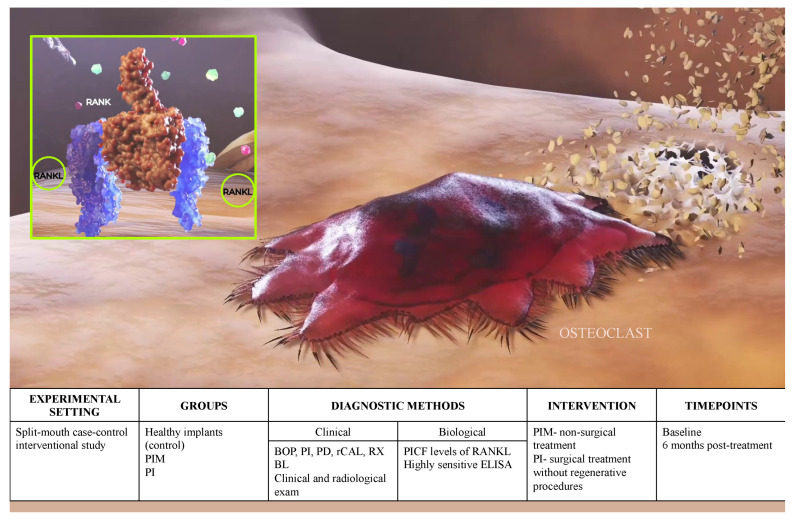
Graphical scheme of the study design.

The present study was designed as a split-mouth interventional study estimating pathological bone resorptions in patients exhibiting peri-implant mucositis (PIM) and peri-implantitis (PI) before and after standard treatments, while the patient represented his own control (Figure 1). Diagnostic capacity of RANKL for peri-implant diagnostics was first estimated according to guidelines for validation of biomarkers [23].

### 2.1. Study Population

Sixty outpatients attending the Clinic for Implantology, Medical Military Academy, Serbia between September 2012 and September 2021 were recruited for the study. They were informed on study procedures and agreed to participate by signing an inform consent form previously approved by the Institutional Ethics Committee of the Medical Military Academy, Belgrade, Serbia (VMA-10-12. A1, 7.11.2011., 23.04.2021.). The study was performed in accordance with the Helsinki declaration [24,25].

### 2.2. Definitions and Study Criteria

Patients presenting at least one implant affected by PIM or one implant affected by PI, and at least one implant with healthy peri-implant tissues at non-adjacent positions, all loaded for at least 2 years, were selected according to the referent case definition [10,26]

- Healthy controls (HC)- bleeding on probing (BOP) ≤ 1/6 points, probing depth < 3 mm and radiological bone loss (RXBL) < 2 mm;
- PIM-BOP > 1/6 sites, PD > 4 mm and RXBL < 2 mm compared to moment of prosthetic loading;
- Peri-implantitis-BOP > 25%, PD > 4 mm and RXBL > 2 mm from the moment of prosthetic loading;
- Treatment success: The absence of deep PPD with BoP/suppuration and no additional bone loss.

The participants were recruited if they were systemically healthy, non-smokers and lacked any of the following exclusion criteria:

- Peri-implant defects requiring regenerative procedures;
- Intake of antibiotics and/or anti-inflammatory agents in the preceding 3 months;
- Previous periodontal treatment in preceding year;
- Aggressive and severe forms of periodontitis;
- Pregnancy and/or lactation in female patients;
- Implant supported restoration with signs of biomechanical overload.

Due to the lack of diagnostic range for measured biomarkers, the split-mouth design was applied to limit potential bias related to inter-individual variability characteristic for BTMs. Additionally, PI cases were restricted to defects not requiring regenerative procedures, to avoid potential impact of biomaterials on local inflammatory profile.

RANKL diagnostic capacity was estimated according to guidelines for biomarker validation; thus the biomarker was requested to be correlated with standard clinical endpoints, with allowed rate of false positivity and false negativity in range of 2.5–10% [23,27].

### 2.3. Clinical Outcome Variables

The full-mouth periodontal measurements were performed using a periodontal probe graded in mm* (North Carolina–Hu-Friedy, Chicago, IL, USA): full-mouth bleeding on probing (FMBoP), full-mouth plaque Index (FMPI), full-mouth probing depth (PD) and full-mouth clinical attachment level (CAL). The clinical examination of all implants was performed using a plastic probe graded in mm * (Colorevue^®^ probe Williams, North Carolina–Hu-Friedy, Chicago, IL, USA) to select the representative site. In case of multiple implants with the same condition, the implant with the worst clinical characteristics in case of disease and the most accessible implant in case of HI was selected as representative. The site-related clinical measurements were recorded in six sites, applying 0.15 N/cm force: BOP, PIi and PD. The RXBL and respective changes from the moment of prosthetic loading were linearly measured on the radiographs [28] being the implant shoulder the referent point. All measurements were performed by two experienced examiners (Z.T. and B.Dj.) after a calibration exercise demonstrating 96.3% concordance within ± 1 mm for measurements of PD.

### 2.4. Anti-Infective Mechanical Treatment (AIMT)

Partially edentulous patients underwent routine non-surgical periodontal treatment including supragingival plaque control and scaling when indicated and were instructed to follow an individualized home care regimen. According to peri-implant pathology the sites were assigned to one of the following treatment protocols:

- PIM: supra + subgingival debridement of the implant surface, implant neck and the abutment for elimination of dental plaque and/or calculus using graphite and/or titanium curettes (Deppeler SA, Rolle, Switzerland). Implant surface was additionally decontaminated using 0.2% chlorhexidine gel (Curasept ADS^®^, Curaprox, Curaden International AG, Kriens, Switzerland);
- PI: open-flap debridement + implant surface decontamination. Briefly, following infiltrative local anesthesia (2% lidocaine with 1:100,000 adrenaline), intrasulcular incisions were created for elevation of buccal and lingual full-thickness flaps. Scaling was performed using graphite and/or titanium curettes, while the chemical implant surface decontamination was performed using gauze soaked infiltrated povidone iodine and 0.2% chlorhexidine gel for 2–3 min each (Curasept ADS^®^, Curaprox, Curaden International AG, Kriens, Switzerland) [29]. The flaps were repositioned and stabilized with interrupted sutures. Postoperative care consisted of rinsing with a 0.12% chlorhexidine gluconate mouthwash (Curasept ADS^®^, Curaprox, Curaden International AG, Kriens, Switzerland) twice a day for 2 weeks, while the sutures were removed 10 days following surgery.

### 2.5. Biomarker Measurement

Diagnostic protocol for biomarker measurement including sampling, storage and analytical protocol were performed as previously described [18,22]. Briefly, the sampling sites were isolated and airdried, while the PICF specimens were collected using filter paper technique [22]. They were later stored in 0.5 mL of sterile phosphate-buffered saline and immediately transferred to the laboratory. PICF samples were collected from the mesial aspect representative implant represented by the implant with the deepest PD in diseased sites and the most accessible implant in the healthy group in case of multiple implants.

The biomarker levels were measured using commercial ELISA kits with the following minimal detection limit, sRANKL: 0.2 pg/mL (Biomedica Gruppe, Vienna, Austria). Biomarker concentrations were expressed as the total biomarker amount (pg/ng) per site in 30 s according to the sampling time method [30].

### 2.6. Data Analysis

The primary outcome variables were PICF levels of sRANKL and their respective changes in response to AIMT. The secondary outcome variables were BOP, PIi, PD and rCAL.

Sample size calculation was performed for the PICF levels of sRANKL estimated in the previous studies [18,22]. According to that, a total sample of 30 participants using α of 0.05 would result in a power of 0.9 with a drop-out of 25%. A pairwise intragroup analysis was performed using Wilcoxon signed-rank test to evaluate the changes in sit-related clinical and biochemical markers. The comparison of the baseline BTMs between HI, PI and PM was assessed using Kruskal–Wallis test, the differences were evaluated using Mann–Whitney test and the *p*-values were adjusted using Bonferroni post hoc test. The correlations between biomarkers and clinical parameters were assessed using Spearman’s rank correlation test in HI and peri-implant diseases. Statistical analysis was performed using commercial software (Prism 5.0, GraphPad Software, Inc., La Jolla, CA, USA).

### 2.7. RANKL Validation for Diagnostic Use

According to guidelines, RANKL was first correlated with standard clinical endpoints, while the accuracy, specificity, sensitivity and respective diagnostic range was estimated using classifying algorithms with decision threshold set to 0.5 as previously described [13,31].

## 3. Results

The final sample comprised of 60 patients and 120 implants without drop-outs between follow-ups. Demographic characteristics including gender (female: 14, 12, male: 16, 18, in PIM and PI, respectively) and age (PIM: 58.2 ± 12.3 and PI: 56.7 ± 14.2) were similarly distributed between the groups. Clinical characteristics of the participants were matched between the groups as well, while respective changes are listed in the Table 1.

### 3.1. Validation of Biomarkers

**Figure 2:**
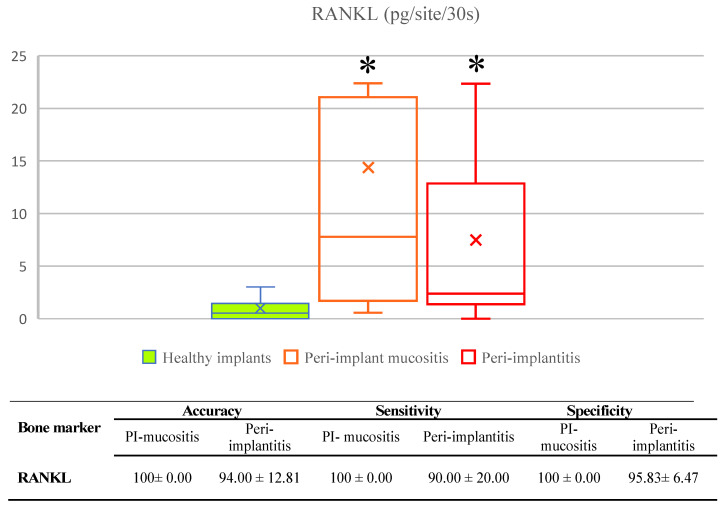
Diagnostic capacity of RANKL for diagnosis of peri-implant diseases. RANKL concentrations were significantly increased in peri-implant mucositis and peri-implantitis when compared to healthy controls, while there was no significant difference between two peri-implant diseases. Based on estimated diagnostic performance, RANKL answered request for diagnostic marker of peri-implant diseases. PI-mucositis-peri-implant mucositis; *-*p* < 0.05; diagnostics performance values are expressed as mean % and standard deviation.

The estimates of diagnostic performance of soluble RANKL for diagnosis of peri-implant diseases is outlined in Figure 2.

RANKL was detectable in all Pima and PI samples as being significantly higher compared to HC, while there was no significant difference in biomarker levels between two peri-implant diseases. RANKL was significantly correlated to all clinical parameters (coefficients: BOP = 0.368, PI = 0.572, PD = 0.401 and rCAL = 0.264; *p* < 0.001). Additionally, RANKL showed high diagnostic performance for diagnosis of both PIM and PI based on high diagnostic accuracy, sensitivity and specificity rates collectively suggesting its suitability as a biomarker bone loss marker in peri-implant diseases.

### 3.2. Effectiveness of Standard Treatment of Peri-Implant Mucositis and Peri-Implantitis

**Figure 3:**
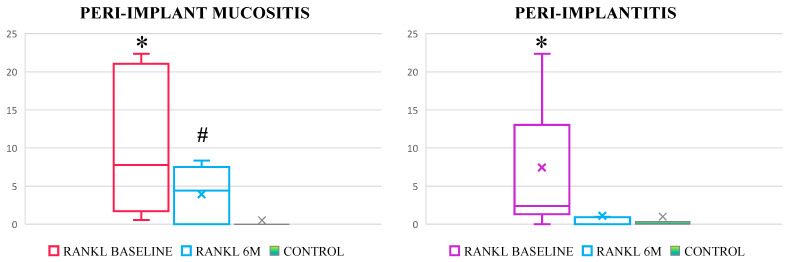
RANKL levels in response to standard treatment of peri-implant mucositis and peri-implantitis. RANKL concentration decreased following treatment in both groups, but respective concentration remained significantly increased in PIM. The boxplots of biomarker estimates are plotted at median values (lines), with respective confidence interval and standard deviations, while the mean values are expressed with markers (x) as well. *-significantly higher compared to post-treatment, *p* < 0.05; #-significantly higher compared to healthy control, *p* < 0.05.

Treatment of both PIM and PI resulted in significant reduction of all clinical parameters, but BOP and PD remained significantly increased in both conditions, as well as rCAL in peri-implantitis at 6 M when compared to HC (Table 1). RANKL levels significantly decreased in both groups, while the RANKL levels remained significantly increased post-treatment in PIM (Figure 3).

## 4. Discussion

Study outcomes showed the capacity of RANKL levels in PICF to reliably reflect active pathological peri-implant bone resorption even before its clinical detectability. The RANKL concentration was significantly increased in both PIM and PI, suggesting the presence of pathological bone resorption in cases that were clinically diagnosed as PIM. Moreover, while the RANKL concentration attained physiological levels following treatment of PI, its concentrations remained significantly increase in PIM, additionally confirming that some clinically diagnosed PIM exhibit PI.

The implant markets size was estimated at 4.6 billion in 2019, with a yearly estimated growth of 9% by 2027 [32]. Given the estimated PI prevalence rate reaching up to 56% [3], the unpredictable responsiveness on standard treatment certainly represents the most devastating aspect of peri-implant diseases. Hence, the treatment of early peri-implant lesions represents the ultimate priority until development of some more predictive protocols [7], while the clinical diagnostics fails to reach requested diagnostic sensitivity on implants [33]. Limitations of clinical diagnosis on implants [34] relate to the implication of wide range of host and implant-related factors being the common reason why modern medicine relies on biomarker-supported diagnostics, since complexity of multifactorial diseases overcome capacity of clinical diagnostics to answer requests of early and precise diagnosis. In the previous study reported by this research team, the active bone resorption has been demonstrated in about 50% PIM patients lacking the clinical signs of bone resorption based on RANKL values comparative to those in peri-implantitis [13], which was confirmed in the present study. The significantly increased RANKL levels in PIM when compared to healthy implants were also confirmed in the recently published study by Chaparro et al. [35]. The trend of RANKL responsiveness to the treatment of peri-implantitis have been demonstrated in a previous study reported by Duarte et al., however the authors were not able to demonstrate statistical significance, most probably due to a very small sample size and low sensitivity of the diagnostic tests [36]. The finding of persistently increased RANKL levels following treatment of PIM confirms that despite reduction of inflammation, standard treatment of PIM fails to provide complete disease resolution [5], additionally confirming that cluster of PIM patients exhibit pathological bone resorption. The mechanism of conversions of PIM into peri-implantitis is unclear [11,37], mostly relating to the facilitated breaching of the soft peri-implant tissues by infection in lack of periodontal fibers, and subsequent uninterrupted spread to the bone. The early diagnosis of peri-implantitis onset and patient compliance to administrated treatment remain of critical importance to halt progression of bone resorption and to minimize the rate of related irreversible tissue destruction proportionally complicating the treatment plan and related costs for timely adjustment of the treatment plan and related to individual patient needs. Again, this imperative ensues from the fact that no single procedure provides predictable outcomes and consistent resolution of peri-implant diseases [5,7]. In the present study, we demonstrated the ability of RANKL to compensate the limitation of clinical diagnosis in the identification of PIM cases with early bone resorption, as well as early non-responsiveness on performed treatment after 6 months. Hence, it is expected that biomarkers can contribute to timely adjustment of the treatment and prescription of more rigorous maintenance strategy with frequent controls in patients with expressed bone resorption. Regarding PI, it was demonstrated that surgical treatment is effective in treatment of moderate defects at the clinical and biomarker levels.

The reason why switching from an “one-size-fit-all” approach to the precision implantology currently represents the leading priority in periodontology and implantology [14] is that this respective approach relies on personalized management strategy built to match the individual patients needs to maximize preventive or treatment effectiveness, good patient perception of the treatment and an optimal cost-effectiveness index [38]. The driving-force of the precision medicine are biomarkers, allowing the clinician to base decisions on more reliable data and to set plans to fit the patient, but so far there is no biomarker validated for the diagnostic use in implantology [39,40]. The major reason for that is limited number of reported diagnostic studies designed according to guidelines for validation of biomarkers for diagnostic use, since majority of biomarker studies are observational cross-sectional studies comparing the levels of different biomolecules between different peri-implant conditions. Validation of the biomarkers is a challenging and very complex process requiring strict adherence to the corresponding recommendations and guidelines, while in the case of implant diagnostics, specifically concerning the PICF, there are additional issues related to the nature of this diagnostic specimens as well, since there is no commercial assay optimized for this kind of specimen. This research team has been working for years on standardization of the diagnostic protocol for biomarker measurement in samples of PICF [18,22,23,41,42]. The present study was designed according to rigorous guidelines for biomarker validation to provide information of highest accuracy regarding capacity of RANKL for precise identification of peri-implant conditions and related patient compliance to administrated treatment within a personalized biomarker-supported concept. According to appraisal criteria, RANKL answered requests for diagnostic markers for peri-implant bone resorption. Additionally, the protocol was lately adjusted for everyday practice by also implementing the time dependent method [30] making it feasible and easy to perform in the clinical setting without supplement equipment. The present study however presents some limitations such as having a relatively small sample size. Hence, although the study was designed according to the sample size calculation and power analysis, studies with larger samples are needed to confirm the present findings.

Thus, a rigorous diagnostic study conducted according to guidelines for biomarker validations, following referent case definitions, with standardized pre-analytical and intra-analytical protocols in the larger sample is urgently needed to accelerate implementation of the biomarker-supported diagnostics in everyday clinical implant practice.

Given the present study outcomes and together with previously reported findings on estimation of the diagnostic performance of clinical parameters for monitoring of peri-implant conditions [34], the biomarker-supported peri-implant diagnostics remains of critical importance for the early diagnosis of peri-implantitis and patient responsiveness on performed treatment. Finally, in the spirit of demonstrated active bone resorption without clinical manifestation following PIM treatment, the use of biomarkers and implementation of personalized approach seems to be option for appropriate progress in resolution of the issue of peri-implant diseases.

## 5. Conclusions

Within the limitations of the study, the cluster of patients lacking the clinical signs of pathological bone loss and exhibiting active bone resorption that persists following standard treatment most probably suggest the early form of peri-implantitis. The biomarker-supported clinical diagnostics on implants seems to be reliable for early identification of pathological bone resorption within clinically inapparent peri-implantitis and in the estimation of the patient compliance to the administrated treatment.

## Figures and Tables

**Table 1:** Clinical characteristics of the study population.

Periodontal Status		Peri-Implant Mucositis		Peri-Implantitis	
Number of teeth (n; mean and range)		17.8 (0–25)		16.4 (5–25)	
Full-mouth PI (% mean ± SD)		25.3 ± 3.9		24.6 ± 4.1	
Full-mouth BOP (% means ± SD)		19.5 ± 4.2		18.39 ± 5.7	
Full-mouth PD (mm; mean ± SD)		4.1 ± 0.7		3.9 ± 1.9	
Full-mouth dental CAL (mm; mean ± SD)		3.2 ± 0.7		3.1 ± 1.2	
**Implant-site parameters**	**Controls**	**Baseline**	**6 months**	**Baseline**	**6 months**
BOP	0	95.25 ± 9.63	29.17 ± 22.13 *^#^	100.0	19.92 ± 3.8 *^#^
PI	21.66 ± 10.51	87.25 ± 28.5	25.7 ± 14.21 *	84.88 ± 25.52	19.87 ± 14.41 *
PD (mm)	1.2 ± 1.57	4.10 ± 1.50	3.25 ± 1.2 *^#^	6.15 ± 1.75	4.28 ± 1.75 *^#^
rCAL (mm)	0	0	0	3.78 ± 1.77	2.67 ± 0.88 ^#^

PI-visible plaque accumulation; BOP-bleeding on probing; PD-probing depth; rCAL-relative clinical attachment level; SD-standard deviation; *-significant decrease compared to baseline value *p* < 0.05, ^#^-significantly higher compared to healthy control.

## Data Availability

The data presented in this study are available on request from the corresponding author. The data are not publicly available due to specific institutional regulations.

## References

[1] Tonetti M.S., Chapple I.L.C., Jepsen S., Sanz M. (2015). Primary and Secondary Prevention of Periodontal and Peri-Implant Diseases: Introduction to, and Objectives of the 11th European Workshop on Periodontology Consensus Conference. J. Clin. Periodontol..

[2] Rakic M., Galindo-Moreno P., Monje A., Radovanovic S., Wang H.-L., Cochran D., Sculean A., Canullo L. (2018). How Frequent Does Peri-Implantitis Occur? A Systematic Review and Meta-Analysis. Clin. Oral Investig..

[3] Romandini M., Lima C., Pedrinaci I., Araoz A., Soldini M.C., Sanz M. (2021). Prevalence and Risk/Protective Indicators of Peri-Implant Diseases: A University-Representative Cross-Sectional Study. Clin. Oral Implants Res..

[4] Figuero E., Graziani F., Sanz I., Herrera D., Sanz M. (2014). Management of Peri-Implant Mucositis and Peri-Implantitis. Periodontology.

[5] Schwarz F., Becker K., Sager M. (2015). Efficacy of Professionally Administered Plaque Removal with or without Adjunctive Measures for the Treatment of Peri-Implant Mucositis. A Systematic Review and Meta-Analysis. J. Clin. Periodontol..

[6] Jepsen S., Berglundh T., Genco R., Aass A.M., Demirel K., Derks J., Figuero E., Giovannoli J.L., Goldstein M., Lambert F. (2015). Primary Prevention of Peri-Implantitis: Managing Peri-Implant Mucositis. J. Clin. Periodontol..

[7] Koldsland O.C., Wohlfahrt J.C., Aass A.M. (2018). Surgical Treatment of Peri-implantitis: Prognostic Indicators of Short-term Results. J. Clin. Periodontol..

[8] Brägger U. (1998). Use of Radiographs in Evaluating Success, Stability and Failure in Implant Dentistry. Periodontology.

[9] Lang N.P., Wetzel A.C., Stich H., Caffesse R.G. (1994). Histologic Probe Penetration in Healthy and Inflamed Peri-implant Tissues. Clin. Oral Implants Res..

[10] Berglundh T., Armitage G., Araujo M.G., Avila-Ortiz G., Blanco J., Camargo P.M., Chen S., Cochran D., Derks J., Figuero E. (2018). Peri-Implant Diseases and Conditions: Consensus Report of Workgroup 4 of the 2017 World Workshop on the Classification of Periodontal and Peri-Implant Diseases and Conditions. J. Clin. Periodontol..

[11] Schwarz F., Derks J., Monje A., Wang H.-L. (2018). Peri-Implantitis. J. Clin. Periodontol..

[12] Romandini M., Berglundh J., Derks J., Sanz M., Berglundh T. (2021). Diagnosis of Peri-Implantitis in the Absence of Baseline Data: A Diagnostic Accuracy Study. Clin. Oral Implants Res..

[13] Rakic M., Monje A., Radovanovic S., Petkovic-Curcin A., Vojvodic D., Tatic Z. (2020). Is the Personalized Approach the Key to Improve Clinical Diagnosis of Peri-Implant Conditions? The Role of Bone Markers. J. Periodontol..

[14] Tonetti M.S., Greenwell H., Kornman K.S. (2018). Staging and Grading of Periodontitis: Framework and Proposal of a New Classification and Case Definition. J. Periodontol..

[15] Carinci F., Romanos G.E., Scapoli L. (2019). Molecular Tools for Preventing and Improving Diagnosis of Peri-Implant Diseases. Periodontology.

[16] Watts N.B. (1999). Clinical Utility of Biochemical Markers of Bone Remodeling. Clin. Chem..

[17] Belibasakis G.N., Bostanci N. (2012). The RANKL-OPG System in Clinical Periodontology. J. Clin. Periodontol..

[18] Rakic M., Lekovic V., Nikolic-Jakoba N., Vojvodic D., Petkovic-Curcin A., Sanz M. (2013). Bone Loss Biomarkers Associated with Peri-Implantitis. A Cross-Sectional Study. Clin. Oral Implants Res..

[19] Theoleyre S., Wittrant Y., Tat S.K., Fortun Y., Redini F., Heymann D. (2004). The Molecular Triad OPG/RANK/RANKL: Involvement in the Orchestration of Pathophysiological Bone Remodeling. Cytokine Growth Factor Rev..

[20] Lacey D.L., Timms E., Tan H.-L., Kelley M.J., Dunstan C.R., Burgess T., Elliott R., Colombero A., Elliott G., Scully S. (1998). Osteoprotegerin Ligand Is a Cytokine That Regulates Osteoclast Differentiation and Activation. Cell.

[21] Yoshinaga Y., Ukai T., Abe Y., Hara Y. (2007). Expression of Receptor Activator of Nuclear Factor Kappa B Ligand Relates to Inflammatory Bone Resorption, with or without Occlusal Trauma, in Rats. J. Periodontal Res..

[22] Rakic M., Struillou X., Petkovic-Curcin A., Matic S., Canullo L., Sanz M., Vojvodic D. (2014). Estimation of Bone Loss Biomarkers as a Diagnostic Tool for Peri-Implantitis. J. Periodontol..

[23] Fleming T.R., Powers J.H. (2012). Biomarkers and Surrogate Endpoints in Clinical Trials. Stat. Med..

[24] (2013). World Medical Association World Medical Association Declaration of Helsinki: Ethical Principles for Medical Research Involving Human Subjects. JAMA.

[25] WMA—The World Medical Association-WMA Declaration of Helsinki—Ethical Principles for Medical Research Involving Human Subjects. https://www.wma.net/what-we-do/medical-ethics/declaration-of-helsinki/.

[26] Sanz M., Chapple I.L. (2012). Working Group 4 of the VIII European Workshop on Periodontology* Clinical Research on Peri-Implant Diseases: Consensus Report of Working Group 4. J. Clin. Periodontol..

[27] Fleming T.R., DeMets D.L. (1996). Surrogate End Points in Clinical Trials: Are We Being Misled?. Ann. Intern. Med..

[28] Van Aken J. (1969). Optimum Conditions for Intraoral Roentgenograms. Oral Surg. Oral Med. Oral Pathol..

[29] de Waal Y.C.M., Raghoebar G.M., Meijer H.J.A., Winkel E.G., van Winkelhoff A.J. (2015). Implant Decontamination with 2% Chlorhexidine during Surgical Peri-Implantitis Treatment: A Randomized, Double-Blind, Controlled Trial. Clin. Oral Implants Res..

[30] Nakashima K., Demeurisse C., Cimasoni G. (1994). The Recovery Efficiency of Various Materials for Sampling Enzymes and Polymorphonuclear Leukocytes from Gingival Crevices. J. Clin. Periodontol..

[31] Quinlan J.R. (2014). C4.5: Programs for Machine Learning.

[32] Dental Implants Market Size & Growth, Industry Report, 2020–2027. https://www.grandviewresearch.com/industry-analysis/dental-implants-market.

[33] Mancini L. (2022). Peri-Implant Health and Diagnostic Considerations. Int. J. Environ. Res. Public. Health.

[34] Monje A., Caballé-Serrano J., Nart J., Peñarrocha D., Wang H.-L., Rakic M. (2018). Diagnostic Accuracy of Clinical Parameters to Monitor Peri-Implant Conditions: A Matched Case-Control Study. J. Periodontol..

[35] Chaparro A., Beltrán V., Betancur D., Sam Y.-H., Moaven H., Tarjomani A., Donos N., Sousa V. (2022). Molecular Biomarkers in Peri-Implant Health and Disease: A Cross-Sectional Pilot Study. Int. J. Mol. Sci..

[36] Duarte P.M., de Mendonça A.C., Máximo M.B.B., Santos V.R., Bastos M.F., Nociti F.H. (2009). Effect of Anti-Infective Mechanical Therapy on Clinical Parameters and Cytokine Levels in Human Peri-Implant Diseases. J. Periodontol..

[37] Fretwurst T., Nelson K., Tarnow D.P., Wang H.-L., Giannobile W.V. (2018). Is Metal Particle Release Associated with Peri-Implant Bone Destruction? An Emerging Concept. J. Dent. Res..

[38] Knight E.T., Thomson W.M. (2018). A Public Health Perspective on Personalized Periodontics. Periodontology.

[39] Duarte P.M., Serrão C.R., Miranda T.S., Zanatta L.C.S., Bastos M.F., Faveri M., Figueiredo L.C., Feres M. (2016). Could Cytokine Levels in the Peri-Implant Crevicular Fluid Be Used to Distinguish between Healthy Implants and Implants with Peri-Implantitis? A Systematic Review. J. Periodontal Res..

[40] Alassy H., Parachuru P., Wolff L. (2019). Peri-Implantitis Diagnosis and Prognosis Using Biomarkers in Peri-Implant Crevicular Fluid: A Narrative Review. Diagnostics.

[41] Rakic M., Petkovic-Curcin A., Struillou X., Matic S., Stamatovic N., Vojvodic D. (2015). CD14 and TNFα Single Nucleotide Polymorphisms Are Candidates for Genetic Biomarkers of Peri-Implantitis. Clin. Oral Investig..

[42] Rakić M., Nikolić-Jakoba N., Struillou X., Petković-Ćurčin A., Stamatović N., Matić S., Janković S., Aleksić Z., Vasilić Đ., Leković V. (2013). Receptor Activator of Nuclear Factor Kappa B (RANK) as a Determinant of Peri-Implantitis. Vojnosanit. Pregl..

